# On the Early Suture of War Wounds

**Published:** 1918-12

**Authors:** 


**On the Early Suture of War-wounds.** By L. SENCERT. *Bull. de la Société de Chirurgie de Paris.* April 31, 1918.

The author of this article disagrees with those who believe that wounds should be sutured within 12 hours after injury, for he has obtained very satisfactory results from sutures performed from 24 to 50 hours after injury.

His hospital once received 56 non-operated war-wounded, whose injuries dated from 24 to 84 hours. Among these were 45 injuries of soft parts, and 11 wounds with fractures.

Among the 45 wounds of soft parts, 20 were occasioned by bullets and presented a very small aperture; they simply received an aseptic dressing. The remaining 35 were widely cleansed, excised, projectiles were removed, and 31 of them were sutured. Among these 28 were healed by first intention.

Among the 11 fractures, 8 were sutured, and 6 of them united by first intention.

The author concludes that suture may be done with success, even for very serious injuries, such as comminutive fractures of the thigh-bone, 48 hours after injury, and 100 kilometers away from the front.

